# 肿瘤相关巨噬细胞：调节肿瘤微环境的新靶点

**DOI:** 10.3779/j.issn.1009-3419.2024.102.13

**Published:** 2024-03-20

**Authors:** Yinxue ZHOU, Dunqiang REN, Huanhuan BI, Bingqian YI, Cai ZHANG, Hongmei WANG, Jiaxing SUN

**Affiliations:** ^1^266000 青岛，青岛大学附属医院呼吸与危重症医学科; ^1^Department of Respiratory and Critical Care Medicine, The Affiliated Hospital of Qingdao University, Qingdao 266000, China; ^2^266000 青岛，青岛大学医学部; ^2^School of Medicine, Qingdao University, Qingdao 266000, China

**Keywords:** 肿瘤相关巨噬细胞, 极化, 抗血管生成治疗, 肺肿瘤, Tumor-associated macrophage, Polarization, Anti-angiogenic therapy, Lung neoplasms

## Abstract

肿瘤相关巨噬细胞（tumor-associated macrophage, TAM）在肺癌免疫微环境中扮演着重要的角色。它们通过自身的表型和吞噬功能的变化，在肺癌的发生和进展中发挥作用，通过促进肺癌免疫抑制型微环境的形成和肿瘤异常血管的生长加速肺癌的侵袭和扩散。巨噬细胞在应对不同刺激时，可以极化为具有不同功能和特征的亚型，分为抗肿瘤M1型和促肿瘤的M2型。在肿瘤组织中，TAM通常极化为交替活化型的M2表型，表现出对肿瘤免疫的抑制作用。本文综述了抗血管生成药物在调节TAM表型方面的作用，它们可以通过将M2型TAM重编程为M1型来发挥抗肿瘤作用。同时，TAM的功能改变在抗血管生成治疗和免疫治疗策略中也起到重要作用。研究证实，TAM通过极化及功能改变可能成为调节肿瘤微环境的新靶点，开辟肺癌治疗的新途径。

## Abstract (en)

Tumor-associated macrophage (TAM) play a crucial role in the immune microenvironment of lung cancer. Through changes in their phenotype and phagocytic functions, TAM contribute to the initiation and progression of lung cancer. By promoting the formation of an immune-suppressive microenvironment and accelerating the growth of abnormal tumor vasculature, TAM facilitate the invasion and metastasis of lung cancer. Macrophages can polarize into different subtypes with distinct functions and characteristics in response to various stimuli, categorized as anti-tumor M1 and pro-tumor M2 types. In tumor tissues, TAM typically polarize into the alternatively activated M2 phenotype, exhibiting inhibitory effects on tumor immunity. This article reviews the role of anti-angiogenic drugs in modulating TAM phenotypes, highlighting their potential to reprogram M2-type TAM into an anti-tumor M1 phenotype. Additionally, the functional alterations of TAM play a significant role in anti-angiogenic therapy and immunotherapy strategies. In summary, the regulation of TAM polarization and function opens up new avenues for lung cancer treatment and may serve as a novel target for modulating the immune microenvironment of tumors.

国际癌症协会发布的数据^[1]^显示，肺癌在全球范围内是导致死亡的主要癌症之一，约占2024年美国癌症病例死亡人群的20.4%，每天约有343人死于肺癌。这一数字几乎是第二大死亡原因结肠癌的2倍，超过了乳腺癌、前列腺癌和胰腺癌的总和。Globocan数据^[2,3]^显示，未来20年肺癌的预测死亡人数将远超胃癌、乳腺癌等常见肿瘤。肺癌的发病率不仅与吸烟等传统因素相关，还受到环境污染和遗传因素的影响。肿瘤相关巨噬细胞（tumor-associated macrophage, TAM）在肺癌免疫微环境中扮演着至关重要的角色。它们通过极化以及改变吞噬功能，既可以促进也可以抑制肺癌的发展^[4,5]^。

巨噬细胞是由骨髓单核细胞分化而来的免疫细胞，它们在机体的免疫防御、抗原提呈、组织稳态维持以及炎症的发生和发展中发挥重要作用^[6]^。在不同的外界刺激下，TAM主要极化为M1或M2表型，其中M1巨噬细胞执行促炎、杀菌和抗肿瘤功能，而M2表型参与抗炎、修复组织和调节免疫平衡。肿瘤微环境（tumour microenvironment, TME）中TAM的M2型表型转变与肺癌的发展密切相关：M2型TAM通过促进肺癌组织内异常血管生成^[7]^、增加肿瘤的耐药性^[8,9]^、促进肿瘤的侵袭和转移^[10]^以及诱导免疫抑制型微环境^[11]^的形成来推动肿瘤进展。

此外，近年来的研究^[12]^表明，调节TAM的功能在肺癌治疗中具有重要的治疗潜力。抗血管生成药物的应用，例如通过将极化为M2型的TAM重编程为M1型，可以发挥抗肿瘤作用。TAM还是抗血管治疗联合免疫治疗以及放化疗发挥作用的重要因素之一^[13]^。因此，通过精确调控TAM的极化和功能，可以为肺癌治疗提供新的策略和方法。

本文综述了TAM在肺癌进展中的作用，包括它们极化与吞噬功能的改变，以及在肺癌抗血管生成治疗中的潜在作用。通过深入了解TAM的生物学特性和与肺癌发展的关系，我们可以更好地理解肺癌的免疫微环境，为开发新的治疗策略提供基础。

## 1 TAM的极化与吞噬功能的改变是肺癌进展的重要机制

### 1.1 TAM表型改变促进肺癌的进展

巨噬细胞主要分为两种表型：促炎M1型和抑炎M2型。因为TAM的表型和功能更接近M2型，因此通常指代分化为M2型的巨噬细胞^[14,15]^。M1型巨噬细胞由脂多糖（lipopolysaccharide, LPS）、干扰素-γ（interferon-γ, IFN-γ）、肿瘤坏死因子α（tumor necrosis factor-α, TNF-α）诱导产生^[15]^，表面标志物包括CD68、CD86以及主要组织相容性复合体II类（major histocompatibility complex II, MHC II）；M1极化后增加诱导型一氧化氮合酶（inducible nitric oxide synthase, iNOS）、TNF-α、CD40、CD86及各种促炎细胞因子如白细胞介素6（interleukin 6, IL-6）、IL-1β、IL-12a、IL-12b的表达，发挥抗肿瘤作用。M2型巨噬细胞表现为免疫抑制表型，由IL-13、IL-4、IL-10、巨噬细胞集落刺激因子（macrophage colony stimulating factor, M-CSF）等诱导产生，根据功能和表型的差异又可进一步分为M2a、M2b、M2c、M2d^[16]^，表面标志物有CD163、CD206，并释放血管内皮生长因子（vascular endothelial growth factor, VEGF）、精氨酸酶1（arginase-1, Arg-1）、IL-10、转化生长因子-β（transforming growth factor β, TGF-β）、吲哚胺2,3-双加氧酶（indoleamine 2,3 dioxygenase, IDO）等，能够促进Th2细胞的激活，有助于肿瘤免疫逃逸、异常血管的生成以及侵袭和转移（图1）^[17,18]^ 。

**图1:**
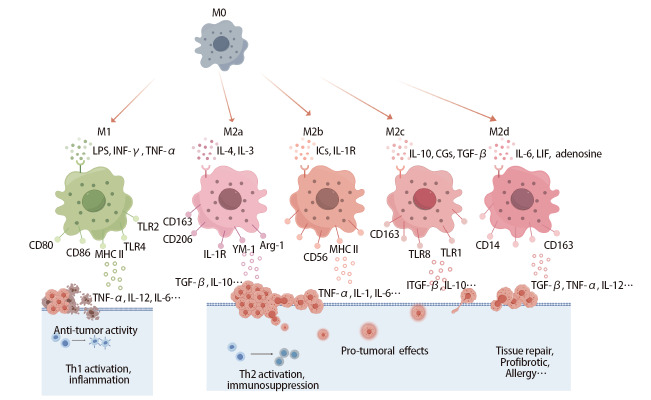
巨噬细胞极化及不同表型巨噬细胞功能示意图。M0在不同细胞因子的刺激下分化为M1、M2a、M2b、M2c、M2d不同的亚型；M1与M2通过分泌不同的细胞因子分别发挥促炎（抗肿瘤）或抗炎（促肿瘤）作用。

M2型TAM与肺癌的不良预后相关^[19]^，在肺癌进展中扮演着重要角色。首先，M2型巨噬细胞释放的抗炎因子如IL-10、TGF-β、人白细胞抗原G（human leucocyte antigen-G, HLA-G）能够抑制T细胞介导的肿瘤杀伤作用，上调免疫抑制程序性细胞死亡配体1（programmed cell death ligand 1, PD-L1）和细胞毒性T淋巴细胞抗原4（cytotoxic T lymphocyte-associated antigen-4, CTLA4）配体表达，从而形成免疫抑制性微环境^[20]^。其次，M2型巨噬细胞分泌的一些促血管生成因子，包括VEGF、血小板源性生长因子（platelet-derived growth factor, PDGF）、表皮生长因子（epidermal growth factor, EGF）、TGF-β、基质金属蛋白酶2（matrix metalloproteinase 2, MMP-2）/MMP-9和其他细胞因子（如TNF-α、IL-1β、IL-8）促进异常的新生血管生成，为肿瘤细胞生长提供营养支持，同时也促进肿瘤细胞的侵袭和转移^[18]^。另外，M2型巨噬细胞还能通过促进TGF-β1的表达来抑制肿瘤细胞的凋亡，从而促进肿瘤的生长和扩散^[21]^。

### 1.2 TAM吞噬功能的改变促进肺癌的进展

巨噬细胞作为固有免疫的重要细胞，可以直接吞噬和杀伤外来病原体、参与肿瘤免疫监测等^[22]^。在肿瘤浸润早期，巨噬细胞主动吞噬肿瘤细胞，但这一现象在后期被肿瘤源性抑制信号抑制^[23]^。CD47信号调节蛋白α（signal regulatory protein alpha, SIRPα）作为被研究最多的抗吞噬通路，阻断CD47-SIRPα之间的识别来诱导增强巨噬细胞对肿瘤细胞的吞噬作用正受到关注^[24]^。如KRAS突变可通过上调CD47的表达协助肿瘤细胞的先天免疫逃逸，阻断KRAS/CD47信号轴可以调节免疫抑制性TME^[25]^。CD47-SIRPα的高表达还促进肺癌治疗中表皮生长因子受体-酪氨酸激酶抑制剂（epidermal growth factor receptor-tyrosine kinase inhibitors, EGFR-TKIs）的获得性耐药，显示了CD47阻断的双重意义^[26]^。此外，许多临床前抗SIRPα的药物正在开发，例如靶向SIRPα的药物联合肿瘤特异性抗体的治疗正显示出优势：Hu5F9-G4联合利妥昔单抗、acc-90002联合利妥昔单抗，ALX148联合曲妥珠单抗（抗Her2）和利妥昔单抗等^[27]^。

## 2 肺癌中调节TAM功能主要分子

TAM免疫调节机制的研究包括集落刺激因子1（colony stimulating factor 1, CSF-1）/集落刺激因子1受体（colony stimulating factor 1 receptor, CSF-1R）轴、IL-4/IL-13与Janus激酶/信号转导因子和转录活化因子6（Janus kinase/signal transducer and activator of transcription 6, JAK/STAT6）传导通路、Toll样受体（Toll-like receptor, TLR）以及CD47-SIRPα信号通路等。CSF-1/CSF-1R轴通过激活磷脂酰肌醇3激酶（phosphatidylinositol-3-hydroxy kinase, PI3K）信号级联反应，调节巨噬细胞的M1/M2极化，影响肿瘤生长和转移；IL-4/IL-13与JAK-STAT6传导通路参与Th2型免疫反应，诱导TAM向M2表型发展，促进肿瘤异常血管生成和进展；TLR通过模式识别病原体相关分子，改变巨噬细胞的激活状态，影响肺癌转移和生长。CD47-SIRPα信号通路通过抑制巨噬细胞介导的吞噬作用，促进肿瘤的生长和转移。这些免疫调节机制的研究为肿瘤治疗提供了新的思路和靶点。

### 2.1 CSF-1/CSF-1R轴

粒细胞-巨噬细胞集落刺激因子（granulocyte-macrophage colony stimulating factor, GM-CSF）主要功能是调节造血细胞的生成和分化，在血管生成过程中也发挥作用。CSF-1与CSF-1R结合，可以通过激活PI3K信号级联反应，进一步促进蛋白激酶B（protein kinase B, AKT）和哺乳动物雷帕霉素靶蛋白复合物2（mammalian target of rapamycin compound 2, mTORC2）的激活，从而调节巨噬细胞的M1/M2极化轴^[28]^。PI3K和AKT激酶的激活或过表达可以抑制M1型巨噬细胞的活化，且PI3K通路的激活介导了可以促进M1生成的核因子-κB（nuclear factor- kappa B, NF-κB）信号通路的负性调节^[29]^。除CSF-1外，CSF-1R还可以与IL-34结合来激活。因此，当IL-34与CSF-1R在肿瘤中高表达时标志着肿瘤进展和更低的生存率。有研究^[30]^证明，由于IL-34和M-CSF高表达的肺癌更易进展至晚期，IL-34和M-CSF及其配体的高表达与肺癌患者队列的低生存率相关。此外，CSF-1可以通过募集和重编程TAM，产生促进肿瘤生长和转移的因子^[31]^。

### 2.2 IL-4/IL-13与JAK-STAT6传导通路

参与Th2型免疫反应的IL-4与IL-13^[32]^是诱导TAM倾向于向促进肿瘤异常血管生成和肿瘤进展的M2表型发展的主要刺激因素之一。IL-13与IL-4通过与I型IL-4受体（IL-4Rα或IL-4Rγc）及II型IL-4受体（IL-4Rα或IL-13Rα1）结合，促进JAK的磷酸化，进而使得转录因子STAT6发生磷酸化，随后，激活的STAT6二聚化并转入细胞核中，与DNA的相应部位结合从而启动靶基因的转录^[4]^。STAT6的激活还可以促进M2相关特异性基因的表达和转录，如Arg-1、甘露糖受体-c1（mannose receptor c type 1, Mrc-1）和参与组织重塑的基因Chil3/Ym1）和抵抗素样分子α（RELM-α或Fizz-1）的表达^[33]^。STAT6作为IL-4与IL-13介导巨噬细胞向免疫抑制表型极化的关键因素，同时也受其他因素的调节。例如有研究^[34]^发现，通过泛素化测定和荧光素酶测定表明，TRAF3可以促进STAT6泛素化和转录活性。位点突变分析显示，STAT6 K450位点泛素化在TRAF3介导的STAT6激活中起至关重要的作用，从而促进了M2相关表面标志物的表达增加以及肿瘤的进展，通过B16黑色素瘤小鼠模型验证了骨髓TRAF3缺乏抑制体内肿瘤生长和肺转移。

### 2.3 TLR

机体对环境的免疫反应可以分为先天免疫和适应性免疫两种，而模式识别受体是先天免疫发挥作用的必经之路^[35]^。TLR是一种跨膜蛋白，是模式识别受体（pattern recognition receptors, PPRs）的一种，可以识别病原体相关的分子模式和损伤相关的分子模式，广泛分布于中性粒细胞、巨噬细胞、树突状细胞、肥大细胞等多种免疫细胞。

截至目前为止，在哺乳动物中一共发现了13种TLR，其中有11种在人体内表达（TLR1-10）^[35]^。巨噬细胞的重编程可以通过不同TLR的激活，由此来改变巨噬细胞的激活状态。例如在肺转移模型中，TLR4可以通过NF-κB通路促进TAM对肺肿瘤转移的作用。通过使用TLR4缺陷的小鼠发现，缺乏TLR4的TAM不能产生促炎的细胞因子，也不产生血管生成因子，并且在肿瘤中无法激活NF-κB的活性，强烈促进肿瘤的生长^[36]^。此外，Lewis肺癌细胞（Lewis lung cancer, LLC）系是最有效的巨噬细胞激活因子，LLC条件培养基能够通过TLR2和TLR6的激活导致巨噬细胞产生TNF-α和IL-6，强烈促进肺癌的转移和生长^[37]^。也有研究^[38]^表明，通过将毒性较小的IFN（包括IFN-α与IFN-β）与TLR激动剂组合应用时可以提高诱导M1型巨噬细胞产生高达100倍的效力，充分展示了其抗肿瘤发展的潜力，并提出基于TLR相关肿瘤免疫治疗的新思路。

### 2.4 CD47-SIRPα

整合素相关蛋白（IAP或CD47）是血小板反应蛋白家族成员的受体，可以调节一系列细胞活性，包括血小板的活化、细胞运动和黏附，白细胞的黏附、迁移和吞噬。CD47是广泛分布于细胞表面的免疫球蛋白，可以抑制巨噬细胞对肿瘤的吞噬作用以促进肿瘤的生长和转移，可以参与介导细胞增殖、迁移、凋亡和免疫稳态^[39]^。SIRPα是一种高表达于膜上的跨膜蛋白，是CD47分子的主要配体，其胞外区域的NH2末端可以与CD47结合，导致免疫受体酪氨酸相关的抑制性基序（immunoreceptor tyrosine-based inhibitory motif, ITIM）上的酪氨酸残基化，使得细胞释放一种抑制吞噬信号，从而可以抑制巨噬细胞介导的吞噬作用，保护机体正常细胞免受免疫系统发挥作用的损伤^[40]^。这种信号传导机制有效地抑制了巨噬细胞介导的吞噬作用，从而保护机体正常细胞不受免疫系统激活时可能造成的损害。CD47已经被证明在各种实体瘤中高表达，并且与肿瘤的不良预后相关，因此，抑制CD47-SIRPα通路，有利于增强机体的适应性免疫反应并提高巨噬细胞的吞噬作用。

在一项对NSCLC患者的分析^[41]^中表明，在2/3的肿瘤样本中存在肿瘤细胞膜上CD47的过表达，癌细胞的CD47过表达与TAM上的SIRPα表达直接相关，表达CD47的癌细胞与SIRPα+Μφ之间存在相互作用。虽然CD68^+^ TAM的存在与良好的预后有关，但当CD68^+^ TAM上存在SIRPα的高表达时，与NSCLC组织中FOXP3^+^肿瘤浸润淋巴细胞（tumor-infiltrating lymphocyte, TIL）的所占比例、低TIL评分、不良预后有直接关联。此外，采用免疫组化方法回顾性分析191例非小细胞肺癌（non-small cell lung cancer, NSCLC）切除患者标本中CD47和PD-L1表达时，发现CD47^+^在女性、非吸烟和肺腺癌患者中表达显著升高（P=0.020、P<0.001和P<0.001），CD47阳性表达与EGFR突变有显著相关性（P<0.001），CD47在NSCLC中的表达与肿瘤PD-L1表达（P=0.0346）和肿瘤突变负荷（P=0.0107）呈负相关^[42]^。

针对EGFR-TKIs获得性耐药的机制研究中发现，在吉非替尼耐药的肿瘤患者标本中观察到TAM的M2样重编程和巨噬细胞吞噬作用的减弱，CD47在耐药的肺癌细胞中上调。通过STAT3抑制剂抑制共培养系统中的CD47-SIRPα信号轴可以增强TAM的吞噬活性，减弱了对EGFR-TKIs的获得性耐药，为克服肺癌治疗中获得性EGFR-TKIs耐药的患者提供了新的治疗策略^[26]^。

## 3 TAM通过分泌免疫抑制性细胞因子及促血管生成促进肿瘤进展

巨噬细胞几乎贯穿了肿瘤生长和进展的整个环节，受TME调节作用的影响，可以重编程为M1或M2表型，M2型巨噬细胞是肿瘤中原发免疫浸润细胞，参与促进肿瘤的侵袭性、生长、血管生成、转移和免疫抑制^[43]^。如M2分泌的外泌体能够通过分泌miR-155和miR-196a-5p直接结合于Ras关联域家族成员3（Ras association domain family member 4, RASSF4）的4'-UTR，负调控RASSF4表达，促进肿瘤细胞的细胞活力、侵袭和迁移能力和上皮间充质转化（epithelial-mesenchymal transition, EMT）^[44]^；同时，肿瘤细胞分泌的外泌体促进巨噬细胞向M2表型的转化以及形成免疫抑制性TME^[45]^。TAM可以通过分泌TGF-β、IL-10、CCL20、MMPs、VEGF以及外泌体等为肺癌的生长和转移提供条件。

TGF-β是调节细胞外基质产生的最关键的细胞因子^[46]^，抑制TGF-β与PD-L1的阻断结合，能够重塑肿瘤胶原基质，增加免疫细胞浸润，充分发挥免疫治疗的优势^[47]^。在肺癌晚期产生的恶性胸腔积液（malignant pleural effusion, MPE）中，由TAM分泌的TGF-β上调T细胞上T细胞免疫球蛋白黏蛋白-3（T cell immunoglobulin and mucin domain 3, TIM-3）、程序性死亡受体1（programmed cell death 1, PD-1）和CTLA-4的表达，诱导T细胞的耗竭和免疫应答的抑制^[48]^。此外，TGF-β作为TME中最主要的炎性因子之一，还参与细胞的EMT和肿瘤细胞的干性^[49]^。

IL-10是一种主要由Th2细胞、B细胞以及单核/巨噬细胞分泌的免疫抑制性细胞因子^[50]^。由M2型巨噬细胞分泌的IL-10具有显著的免疫抑制作用，能够促进Th2细胞的激活，有助于肿瘤的免疫逃逸^[17]^。在对63例患者的原发肺肿瘤组织分析中发现，TAM表达高水平的IL-10与肿瘤分期、肿瘤大小、淋巴结转移、淋巴血管浸润与组织学分化不良显著相关^[51]^。TAM分泌的IL-10还可以通过JAK1/STAT1/NF-κB/Notch1信号传导促进NSCLC的癌症干细胞（cancer stem cell, CSC）样特性，阻断IL-10/JAK1信号传导，可以抑制TAM介导的NSCLC肿瘤在体内的生长，以及TAM介导的NSCLC中CSC相关和间充质相关基因的表达。因此，IL-10/JAK1信号传导可能成为NSCLC治疗的潜在治疗靶点^[52]^。

趋化因子是一类由肿瘤或免疫细胞分泌的介导趋化和转移的可溶性细胞因子，包括CCL5、CCL20、CXCL14等，他们可通过与其同源蛋白结合在促进肿瘤进展中共同发挥作用。在研究人NSCLC标本中单核细胞浸润情况时发现NSCLC肿瘤组织中趋化因子的水平高于正常肺组织^[53]^。此外，在NSCLC组织中明显存在CCL20的过表达，利用A549研究发现，CCL20的分泌可以通过激活ERK和PI3K通路促进肺癌的迁移和增殖^[54]^。CCL2中和治疗可以抑制肺癌的转移以及提高患者的生存率，但停止CCL2的治疗会加速小鼠的肺转移和死亡^[55]^。

MMPs代表与肿瘤发生相关性最强的蛋白酶家族，除可以通过细胞外基质降解促进肿瘤的迁移外，还可以调控细胞生长、调节炎症反应和血管生成相关信号通路^[56]^。在一项通过多重免疫测定法评估57例晚期NSCLC患者的MMPs血清浓度研究^[57]^中，与35例慢性阻塞性肺疾病（chronic obstructive pulmonary disease, COPD）患者的血清样本相比，NSCLC的循环MMP-8和MMP-12水平更高。利用从NSCLC中分离的TAM与3种肺癌细胞系共培养后，发现TAM可以通过上调肿瘤细胞中MMP-9的表达来促进肺癌的侵袭性^[58]^。

TAM是TME中血管生成环节的关键细胞，是促血管生成因子和细胞外基质降解介质的主要来源，包括VEGF、EGF、PDGF、TGF-α/-β、血管生成素1/2（angiopoietin 1/2, Ang-1/-2）、MMPs（例如MMP-2、MMP-9和MMP-12）和丝氨酸或半胱氨酸蛋白酶等^[59]^。巨噬细胞的M2极化显著增强VEGF-A和VEGF-C在蛋白层面和mRNA层面的表达，与异常血管生成显著相关，是NSCLC患者预后不良的有力指标^[60]^。除此之外，VEGF也是诱导TAM产生和聚集的关键分子，可以与TAM分泌产生的VEGF形成一个正反馈环，进一步促进了肿瘤的血管生成^[61]^。M2型巨噬细胞分泌的外泌体可以被肺腺癌细胞吸收，这种含有miRNA的外泌体可以促进肺腺癌细胞的侵袭和迁移，并促进肿瘤的异常血管生成^[62]^。体内实验研究^[59]^表明，TAM靶向治疗与抗血管生成治疗联合使用可以达到协同增效的效果，因此为了增加抗血管治疗的效果，应在靶向血管生成治疗的同时，与TAM的重编程结合起来。

## 4 TAM在肺癌抗血管生成治疗中的作用

尽管肺癌有相当一部分在肿瘤免疫分型中属于“免疫沙漠型肿瘤”，但肿瘤组织中仍有相当数量的巨噬细胞浸润（约占总白细胞的20%）^[63]^，抗血管生成治疗可以通过调节TAM的功能以及重编程抑制肿瘤生长和转移。TAM作为肺癌微环境中发挥抗肿瘤作用的重要角色，正在受到临床广泛关注（表1^[64⇓⇓⇓⇓⇓⇓⇓⇓-73]^）。

**表1:** 肺癌相关巨噬细胞的临床试验

Medicine	Type	Clinical phase	Intervening measure	Ref.
Sintilimab, SBRT and GM-CSF	Advanced NSCLC	II	SBRT to one lesion, followed by Sintilimab and GM-CSF within 2 weeks after SBRT	^[64]^
Camrelizumab combined with Apatinib	Advanced nonsquamous NSCLC	II	Patients received Apatinib in combination with Camrelizumab	^[65]^
RRx-001, Etoposide, Cisplatin	Relapsed SCLC	II	Patients received 4 mg RRx-001 once weekly until progression followed by the start of EP (Etoposide platinum)	^[66]^
Atezolizumab	NSCLC	II	Chemoradiation and Atezolizumab	^[67]^
Imalumab (BAX69)	NSCLC	I	Imalumab (BAX69)	^[68]^
Nivolumab	Resected NSCLC	/	Nivolumab	^[69]^
Gefitinib or Erlotinib	Advanced lung adenocarcinoma	/	Received 2-4 cycles of Platinum-doublet chemotherapy and diseases progressed before the treatment of EGFR-TKIs	^[70]^
Gemcitabine, Docetaxel, GM-CSF, Aldesleukine	NSCLC	Ⅱ	Biweekly Gemcitabine and Docetaxel +/- GM-CSF and low dose Aldesleukine	^[71]^
KRN7000-pulsed IL-2/GM-CSF-cultured PBMCs	Advanced and Recurrent NSCLC	I-II	Patients received a total of four i.v. injections of αGalCer-pulsed IL-2/GM-CSF-cultured PBMCs	^[72]^
Vaccination with irradiated autologous tumor cells	Metastatic NSCLC	I	Resected metastases were processed to single-cell suspension, infected with a replication-defective adenoviral vector encoding GM-CSF	^[73]^

NSCLC: non-small cell lung cancer; SBRT: stereotactic body radiotherapy; GM-CSF: granulocyte-macrophage colony stimulating factor; IL-2: interleukin-2; PBMCs: peripheral blood mononuclear cells; EGFR-TKIs: epidermal growth factor receptor-tyrosine kinase inhibitors.

肺癌组织中的异常血管不仅为肿瘤细胞提供了营养物质，还通过损害灌注造成局部缺氧和降低pH值的微环境，从而促进了肺癌的恶化。常见的促进血管生成的生长因子包括EGF家族、EGFR、成纤维细胞生长因子（fibroblast growth factor, FGF）、CSF、人表皮生长因子受体2（human epidermal growth factor receptor 2, HER2）和HER3以及PDGF等^[74]^。通过抗血管生成药物调节TAM的功能，促进TAM的重编程，诱导TAM由M2表型向M1转化，能够有效抑制肿瘤生长和转移，改善肺癌患者预后，为患者生存率的提高带来新希望。

近年来，体内外实验证实有多种药物可以阻止TAM向M2的极化或将M2重编程为M1。常用的多靶点血管生成抑制剂如阿帕替尼处理与A549或H1299条件培养基共培养4 h的人单核白血病细胞THP-1之后，Western blot或聚合酶链式反应（polymerase chain reaction, PCR）检测巨噬细胞表面PD-L1的表达下调，表明阿帕替尼对免疫抑制性TME的正向调节作用^[75]^；此外，低剂量的阿帕替尼更容易抑制髓源性抑制细胞（myeloid-derived suppressor cell, MDSC）的募集以及TAM表面M2型标志物CD163的表达^[76]^，还可以抑制巨噬细胞介导的EMT以及肺肿瘤细胞的迁移^[77]^。抗VEGF治疗时，同时阻断由TNF-α/NF-κB通路介导的CD47分子上调，不仅能够增强巨噬细胞的吞噬作用，还增强抗VEGF治疗的效果，有利于杀伤肿瘤的巨噬细胞的激活^[78]^。此外，多靶点药物安罗替尼能够增强M1型巨噬细胞的浸润，显著降低M2型TAM的百分比^[77]^。内皮抑素发挥的抗血管生成和抗肿瘤作用通过阻断P38 MAP激酶和Erk1/2信号通路阻断巨噬细胞的迁移和替代活化，抑制TAM诱导的肿瘤细胞增殖和血管密度^[79]^。

TAM也是抗血管联合免疫治疗、放化疗等协同发挥作用的关键因素之一。尽管放化疗对局部肿瘤控制至关重要，但某些肿瘤不敏感甚至促进生长和侵袭，与TME中巨噬细胞的变化密切相关。通过调控诸如CSF-1/CSF-1R和CXCL12/CXCR4等信号通路，可以影响巨噬细胞在放疗中的作用，从而影响肿瘤的治疗效果^[80]^。此外，靶向VEGFR2和MHC I类链相关分子的双特异性融合蛋白mAb04-MICA可以诱导TAM在体外和体内由M2向M1极化的同时，应用抗PD-1抗体可以通过刺激自然杀伤（natural killer, NK）细胞和CD4^+^ T细胞活化增强其抗肿瘤效果^[13]^。因此，抗血管生成治疗通过抑制免疫抑制型巨噬细胞的生成，以时间和剂量依赖性调节肿瘤血管的正常化，使得肿瘤细胞表面表达的PD-L1上调，增强了抗PD-1和PD-L1治疗的敏感性^[81]^。

通过一系列的体内和体外实验证明，抗血管生成治疗对于调控肺癌微环境中的巨噬细胞功能具有重要意义，其在肿瘤治疗中的作用日益受到重视。通过调节巨噬细胞的功能和重编程，抗血管生成治疗为肺癌患者带来了新的治疗机遇，为改善肿瘤预后和提高患者生存率提供了新的希望。

## 5 总结与展望

综上所述，TAM在肺癌的进展中扮演着至关重要的角色。它们可以通过极化为具有免疫抑制功能的M2表型以及降低吞噬能力等机制促进肺癌的发展。肿瘤组织通过分泌CSF-1、IL-13、IL-4等细胞因子促进TAM的M2型极化，从而形成免疫抑制型的TME。相应地，极化的TAM可以通过分泌TGF-β、IL-10、CCL20、MMPs等促进肺癌的侵袭和进展。

TAM的重编程被认为是抗血管生成治疗药物发挥作用的关键因素之一。这些药物通过改变TAM的极化状态，不仅可以抑制肿瘤生长，还能增强其他治疗方法如免疫治疗的治疗效果。然而，如何更有效地将调节巨噬细胞功能与其他治疗手段相结合，以最大程度地发挥它们在免疫微环境中的作用，仍然是一个挑战。当前，对TAM在肺癌治疗中的作用机制的了解还远未完全，需要更多的实验和临床研究来深入探索。

对未来的研究方向而言，探索新的药物和治疗方法来调节TAM的活性和功能，尤其是在抗血管生成治疗领域，将是一个重要的研究焦点。由于单一治疗方式的局限，靶向TAM的同时联合抗血管生成治疗、免疫治疗和放化疗可能会成为弥补缺陷更好的方式。TAM在免疫抑制微环境中的作用以及其与PD-L1的相互关系对于肺癌治疗的重要性不言而喻。深入研究TAM如何调节肿瘤免疫抑制微环境的形成和维持以及其在PD-L1表达调控中的作用机制，为设计新型的联合治疗策略提供了宝贵的线索。同时，深入了解TAM与其他TME中细胞的相互作用，将为更精准的治疗方案设计提供有益参考，为肿瘤治疗领域带来新的突破。
